# Denosumab-induced hypocalcemia in patients with solid tumors and renal dysfunction: a multicenter, retrospective, observational study

**DOI:** 10.1186/s12885-024-11942-2

**Published:** 2024-02-15

**Authors:** Kazuyo Nakamura, Michihiro Kaya, Yuki Yanagisawa, Keisuke Yamamoto, Nana Takayashiki, Hirotoshi Ukita, Mariko Nagura, Kaori Sugiue, Mariko Kitajima, Kumi Hirano, Hiroki Ishida, Chiharu Onoda, Yutaka Kobayashi, Eiji Nakatani, Keiichi Odagiri, Takaya Suzuki

**Affiliations:** 1 Shizuoka General Hospital, -27-1, Kita-ando, Aoi-ku, 420-8527 Shizuoka, Japan; 2 Shizuoka Cancer Center, Nagaizumi, Japan; 3 Seirei Hamamatsu General Hospital, Hamamatsu, Japan; 4 Iwata City Hospital, Shizuoka, Japan; 5 Chutoen General Medical Center, Kakegawa, Japan; 6 Japanese Red Cross Shizuoka Hospital, Shizuoka, Japan; 7 Shizuoka City Shizuoka Hospital, Shizuoka, Japan; 8 Hamamatsu Medical Center, Hamamatsu, Japan; 9 JA Shizuoka Kohseiren Enshu Hospital, Hamamatsu, Japan; 10 Yaizu City Hospital, Yaizu, Japan; 11 Fujinomiya City General Hospital, Fujinomiya, Japan; 12 Shizuoka Graduate University of Public Health, Shizuoka, Japan; 13 Center for Clinical Research, Hamamatsu University Hospital, Hamamatsu, Japan

**Keywords:** Denosumab, Bone metastases, Renal insufficiency, Hypocalcemia

## Abstract

**Background:**

Bone metastases are frequently observed in advanced cancer, and bone modifying agents are used to prevent or treat skeletal-related events. Zoledronic acid is contraindicated in patients with severe renal impairment (Ccr < 30 mL/min), but it is not completely known whether denosumab can be used in them. We aimed to determine the association between renal function and hypocalcemia development during denosumab treatment.

**Methods:**

We included patients with solid cancer and bone metastases who started denosumab treatment between April 2017 and March 2019. They were classified into four groups based on creatinine clearance (Ccr; mL/min): normal (Ccr ≥ 80), mild (50 ≤ Ccr ˂80), moderate (30 ≤ Ccr ˂50), and severe (Ccr ˂30). Hypocalcemia was evaluated using the Common Terminology Criteria for Adverse Events (v5.0) based on the albumin-adjusted serum calcium levels; its incidence (stratified by renal function) and risk factors were investigated using a Chi-square test and logistic regression analysis.

**Results:**

Of 524 patients (age: 69 ± 11 years; 303 men), 153 had a normal renal function and 222, 117, and 32 had mild, moderate, and severe renal dysfunction. The albumin-adjusted serum calcium level was higher than the measured (total) calcium level in most patients. The incidence of grade ≥ 1 hypocalcemia was 32.0% in the normal group and 37.4%, 29.9%, and 62.5% in the mild, moderate, and severe renal dysfunction groups, respectively. It was, therefore, higher in the severe renal dysfunction groups than in the normal group (*P* = 0.002). The incidence of grade ≥ 3 hypocalcemia did not differ significantly among the groups. Pre-treatment low serum calcium levels and severe renal dysfunction were risk factors for hypocalcemia.

**Conclusions:**

Evaluating denosumab-induced hypocalcemia required albumin adjustment, and its incidence was high among patients with severe renal dysfunction. Reduced serum calcium levels and severely impaired renal function were associated with an elevated hypocalcemia risk.

**Supplementary Information:**

The online version contains supplementary material available at 10.1186/s12885-024-11942-2.

## Background

The prevalence of bone metastasis in advanced cancer is 65–75% for breast and prostate cancer, 30–40% for lung cancer, 40% for bladder cancer, 20–35% for renal cancer, and 5% for digestive system cancer [1, 2]. Patients with cancer often develop renal dysfunction due to cancer progression and treatment; related conditions include chronic kidney disease (CKD), direct tumor invasion, tumor lysis syndrome, and adverse events induced by anticancer drugs [3]. There is a high prevalence of kidney injury in patients with cancer (12–20%), 1.3% of whom are reported to have a creatinine clearance (Ccr) of < 30 mL/min [4–5].

Zoledronic acid, a bisphosphonate, has been used to treat bone metastases of cancers. It requires dose adjustment in patients with renal impairment and is contraindicated in patients with severe renal impairment (Ccr < 30 mL/min) [6]. In contrast, denosumab, a human anti-nuclear factor kappa B ligand-activating antibody, was reported to be useful for bone metastases and is now in common use [7–8]. The risk of skeletal-related events was reduced by 17% with denosumab compared with zoledronic acid [9]. Renal dysfunction does not affect the pharmacokinetics or pharmacodynamics of denosumab; hence, it does not require dose adjustment based on the renal function [10]. However, it is not completely known whether denosumab can be used for the treatment of bone metastases in patients with severe renal dysfunction in whom zoledronic acid is contraindicated.

On the other hand, the incidence of hypocalcemia as an adverse event is higher with denosumab than with zoledronic acid [9], and represents the most significant clinical problem. In particular, denosumab-induced hypocalcemia is often overlooked in patients with renal dysfunction. This is because renal failure reduces the concentration of albumin in the blood; since albumin binds to calcium, its reduction makes calcium levels appear high. To date, no study has clarified the differences in the frequencies of true hypocalcemia (adjusted for albumin) and unadjusted hypocalcemia.

Therefore, we evaluated the incidence of hypocalcemia, according to the renal function level and with and without albumin adjustment, in patients treated with denosumab for bone metastases from solid tumors. In addition, the purpose of this study was to determine the exact risk factors for hypocalcemia.

## Methods

### Study design and population

We conducted this multicenter, retrospective, cohort study to investigate the relationship between renal function and the incidence of denosumab-induced hypocalcemia in patients with solid cancers. This clinical study was conducted at 13 hospitals in the Shizuka Prefecture, Japan. Patients who were newly administered with denosumab for metastatic bone lesions caused by solid cancer between April 2017 and March 2019 were analyzed retrospectively. Patients who did not undergo blood testing 8 weeks after starting denosumab therapy were excluded from the study.

### Pretherapeutic variables

Data on patient characteristics (age, sex, height, body weight, body surface area, cancer type, and dialysis [y/n]), laboratory test results (serum levels of calcium, inorganic phosphorus, albumin, and creatinine), and concomitant drugs (oral supplementation of calcium and vitamin D and other medications that affect calcium levels) were collected by reviewing medical records.

### Determination of renal function levels using estimated Ccr

Renal function was evaluated in terms of the Ccr value (estimated using the Cockcroft–Gault formula [11]), and the patients were divided into the following four groups: (1) normal renal function (Ccr, ≥ 80 mL/min), (2) mild renal dysfunction (Ccr, 50–80 mL/min), (3) moderate renal dysfunction (Ccr, 30–49 mL/min), and (4) severe renal dysfunction (Ccr, < 30 mL/min or undergoing dialysis) [10, 12]. When the body weight exceeded the ideal weight or was unknown within 4 weeks of starting denosumab treatment, the estimated Ccr was calculated using ideal body weight (IBW). The IBW was calculated using the following formula:

$$IBW{\rm{ }} = {\rm{ }}{\left( {Height{\rm{ }}\left[ m \right]} \right)^2} \times {\rm{ }}22$$

Accordingly, the renal function level-specific hypocalcemia incidence was investigated.

### Hypocalcemia incidence

The primary outcome of this study was the incidence of renal function-associated hypocalcemia. The patients were followed from the first dose until the second dose (8 weeks after the first dose if not administered within 8 weeks after the first dose).

Hypocalcemia was defined as grade ≥ 1 using the albumin-adjusted calcium concentration according to the U.S. NCI Common Terminology Criteria for Adverse Events version 5.0 [13] or as the initiation, increase, or change in the calcium or vitamin D supplementation. The patients were followed from the first dose until the second dose (8 weeks after the first dose if not administered within 8 weeks after the first dose).

Serum albumin was measured alongside serum calcium levels (without albumin adjustment), and the serum calcium and albumin-adjusted calcium concentrations were calculated. If the albumin level was < 4 g/dL, the albumin-adjusted calcium concentration was calculated using the following formula:

Adjusted calcium concentration [mg/dL] = measured calcium concentration [mg/dL]) + 4– albumin [g/dL])

### Statistical analysis

Continuous variables are presented as mean and standard deviation, while categorical variables are presented as frequency (percentage). Between the normal renal function group and the renal dysfunction groups, continuous variables were compared using the Kruskal–Wallis test and categorical variables were compared using the chi-squared test. Linear regression analysis was performed to assess the relationship between the serum albumin levels and renal dysfunction. A paired t-test demonstrated the difference between the measured (total) calcium and albumin-adjusted calcium levels. Risk factors for hypocalcemia were investigated using univariate and multivariate logistic regression analyses, with patient characteristics and concomitant medication usage as the explanatory variables. The odds ratios, 95% confidence intervals, and *P*-values were calculated. Variables with *P* < 0.05 in the univariate analysis were analyzed in the multivariate model, and variables with *P* < 0.05 in the multivariate model were identified as risk factors. Because this study was exploratory, no adjustment for the multiplicity of tests was carried out. All statistical analyses were performed using the EZR (Easy R) software [14]. Statistical significance was determined using two-sided tests with P ˂0.05.

### Ethics

The study protocol complied with the Declaration of Helsinki and the Japanese Ethical Guidelines for Medical and Health Research Involving Human Subjects. It was approved by the Ethics Committee of the Shizuoka General Hospital (approval no.: SGHIRB#2,019,066). The committee waived the requirement of informed consent because the study represented a retrospective observational analysis. Instead, the use of an “opt-out (by public notice)” approach to consent was approved. A written explanation of data from clinical investigations was provided on the hospitals’ websites. Patients did not provide written informed consent but were allowed to decline participation.

## Results

### Study population

Between April 2017 and March 2019, 673 patients were prescribed denosumab for solid tumors. Among these, 149 patients were excluded for the following reasons: missing baseline renal function data (*n* = 1), missing calcium data (*n* = 44), missing albumin data (*n* = 67), and missing follow-up data (*n* = 37). Finally, the current analysis was restricted to 524 patients (303 men and 221 women).

### Patient characteristics

Patient characteristics, classified by renal function, are shown in Table 1. The age of all patients ranged from 34 to 96 years with the mean of 68.7 ± 11.3 years. The most common type of cancer was lung cancer (42%), followed by breast cancer (18%) and prostate cancer (17%). Approximately 90% of all patients were administered a concomitant supplement to prevent hypocalcemia, comprising native vitamin D with or without calcium (441 patients; 84%), activated vitamin D with or without calcium (27 patients; 5%), and calcium alone (1 patient; 0.2%).

**Table 1:** Patient characteristics classified by renal function

Variable	Renal function classification				*P*-value	Total (*n* = 524)
	Normal	Mild	Moderate	Severe		
	(*n* = 153)	(*n* = 222)	(*n* = 117)	(*n* = 32)		
Age (years)	59.0 ± 10.3	70.5 ± 8.3	75.6 ± 8.6	78.0 ± 10.2	< 0.001	68.7 ± 11.3
Sex, male/female (n)	77/76	135/87	69/48	22/10	0.119	303/221
Body weight (kg)	60.3 ± 11.9	55.9 ± 10.3	50.9 ± 11.4	49.4 ± 12.2	< 0.001	55.7 ± 11.8
	(*n* = 142)	(*n* = 191)	(*n* = 99)	(*n* = 30)		(*n* = 462)
Serum creatinine (mg/dL)	0.60 ± 0.12	0.76 ± 0.17	1.00 ± 0.27	2.14 ± 1.23	< 0.001	0.85 ± 0.50
Creatinine clearance (mL/min)	97.8 ± 14.4	64.5 ± 8.4	41.5 ± 5.6	21.2 ± 6.0	< 0.001	66.4 ± 25.6
Serum calcium level (mg/dL)	9.0 ± 0.6	9.2 ± 0.8	9.4 ± 1.1	9.9 + 2.3	0.358	9.2 ± 1.0
Albumin-adjusted serum calcium level (mg/dL)	9.6 ± 0.6	9.6 ± 0.8	10.1 ± 1.2	10.9 + 2.4	< 0.001	9.8 ± 1.1
	(*n* = 149)	(*n* = 218)	(*n* = 113)	(*n* = 32)		(*n* = 512)
Serum albumin (g/dL)	3.6 ± 0.7	3.6 ± 0.7	3.3 ± 0.6	3.1 ± 0.7	< 0.001	3.5 ± 0.7
	(*n* = 149)	(*n* = 218)	(*n* = 113)	(*n* = 32)		(*n* = 512)
Serum phosphorus (mg/dL)	3.3 + 0.8	3.4 + 0.7	3.3 + 0.6	3.8 ± 0.8	0.108	3.4 ± 0.7
	(*n* = 68)	(*n* = 94)	(*n* = 59)	(*n* = 23)		(*n* = 244)
Cancer type Lung cancer	61 (40)	93 (42)	52 (44)	13 (41)	0.084	219 (42)
Breast cancer	36 (24)	41 (18)	13 (11)	2 (6)		92 (18)
Prostate cancer	17 (11)	42 (19)	21 (18)	8 (25)		88 (17)
Other	39 (25)	46 (21)	31 (26)	9 (28)		125 (24)
Prophylactic administration						
Natural vitamin D ± calcium	137 (90)	193 (87)	92 (79)	19 (59)	< 0.001	441 (84)
Active vitamin D ± calcium	7 (5)	7 (3)	6 (5)	7 (22)		27 (5)
Calcium only	0 (0)	0 (0)	0 (0)	1 (3)		1 (0.2)
None	9 (6)	22 (10)	19 (16)	5 (16)		55 (10)

Values in parentheses represent percentages

Among the 524 included patients, 153 (29%) had normal renal function, while 222 (43%), 117 (22%), and 32 (6%) had mild, moderate, and severe renal dysfunction, respectively. Decreased renal function was associated with higher age, lower body weight, and lower serum albumin levels.

### Serum calcium levels (without albumin adjustment) and albumin-adjusted calcium levels according to the renal function levels

**Fig. 1:**
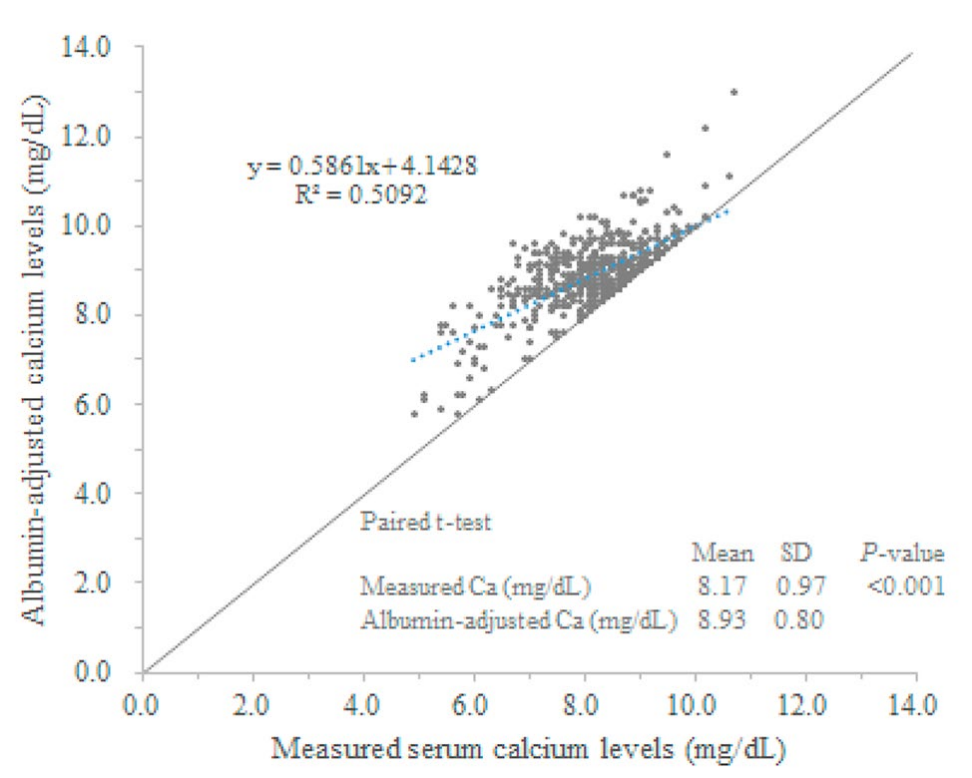
Correlation between albumin-adjusted calcium levels and measured serum calcium levels1^2^

Serum calcium levels remained constant regardless of the renal function level (*P* = 0.358, Table 1). In contrast, the serum albumin levels decreased (*P* < 0.001) and albumin-adjusted serum calcium levels increased (*P* < 0.001) with the degree of renal dysfunction. In addition, as shown in Fig. 1, the level of albumin-adjusted serum calcium was higher than the measured serum calcium level in most patients (*P* < 0.001).

### Differences in hypocalcemia incidence according to the serum calcium levels (without albumin adjustment) and albumin-adjusted serum calcium levels

When assessed according to the serum calcium levels (without albumin adjustment), the incidence of grade ≥ 1 and ≥ 3 hypocalcemia were 69.3% (*n* = 363) and 10.7% (*n* = 56), respectively. When assessed according to the albumin-adjusted serum calcium levels, the incidence of grade ≥ 1 and ≥ 3 hypocalcemia decreased to 35.7% (*n* = 187) and 2.5% (*n* = 13), respectively.

**Fig. 2:**
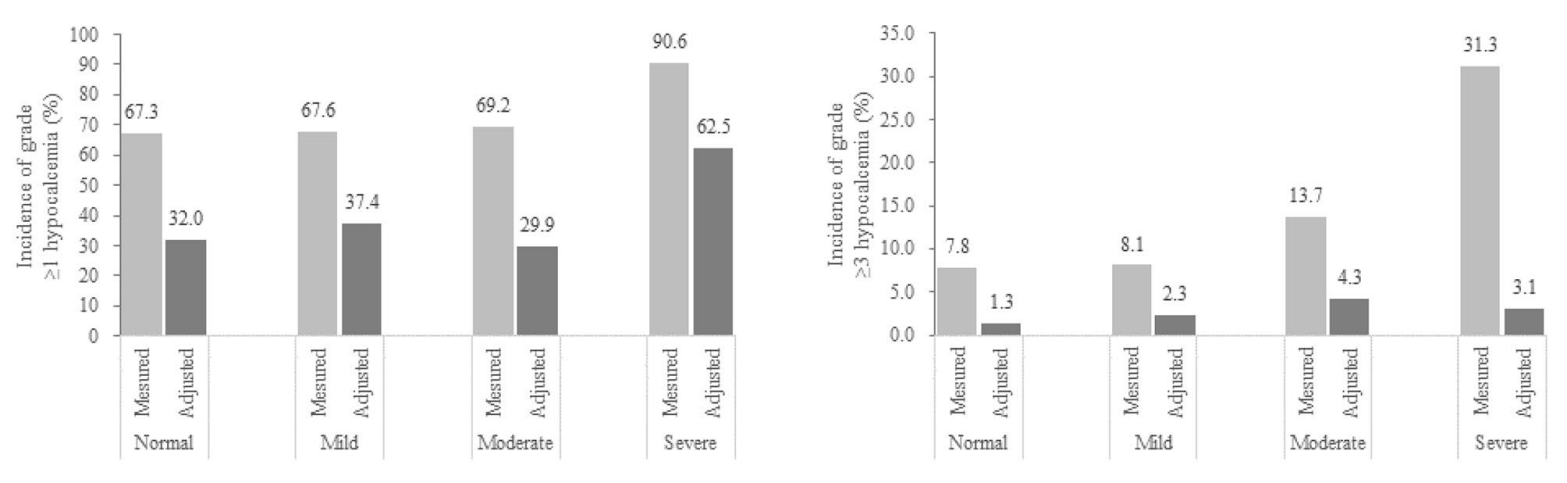
Hypocalcemia incidences by renal function

The hypocalcemia incidences by renal function are shown in Fig. 2. The incidence of hypocalcemia was lower with albumin-adjusted serum calcium levels than with measured serum calcium levels (without albumin adjustment). When the calcium level was adjusted for albumin, the incidence of grade ≥ 1 hypocalcemia was found to be higher in the severe renal dysfunction group than in the normal renal function group (*P* = 0.002); however, the incidence of grade ≥ 3 hypocalcemia did not differ between the two groups (*P* = 0.436).

### Risk factors for denosumab-induced hypocalcemia

The characteristics of patients with and without hypocalcemia are shown in Table 2. The results of the univariate logistic regression analysis are shown in Supplementary Table 1. Supplement usage and cancer type were not identified as significant risk factors.

**Table 2:** Characteristics of patients with and without hypocalcemia

		Patients without hypocalcemia (*n* = 337)	Patients with hypocalcemia (*n* = 187)	*P*-value
Age (years)		68.8 ± 11.4	68.6 ± 11.0	0.542
Sex, male/female (n)		188/149	115/72	0.230
Body weight (kg)		55.5 ± 11.9 (*n* = 300)	56.3 ± 11.6 (*n* = 162)	0.294
Serum creatinine (mg/dL)		0.80 ± 0.38	0.94 ± 0.66	0.009
Creatinine clearance (mL/min)		67.9 ± 25.2	63.7 ± 26.0	0.108
Renal function classification	Normal	104 (31)	49 (26)	0.006
	Mild	139 (41)	83 (44)	
	Moderate	82 (24)	35 (19)	
	Severe	12 (4)	20 (11)	
Serum calcium level before administration of denosumab (mg/dL)		9.3 ± 0.9	9.0 ± 1.1	< 0.001
Albumin-adjusted serum calcium level before administration of denosumab (mg/dL)		9.9 ± 1.0 (*n* = 328)	9.6 ± 1.1 (*n* = 184)	< 0.001
Serum albumin (g/dL)		3.5 ± 0.7 (*n* = 328)	3.5 ± 0.6 (*n* = 184)	0.883
Serum phosphorus (mg/dL)		3.4 ± 0.7 (*n* = 139)	3.4 ± 0.7 (*n* = 105)	0.611
Cancer type	Lung cancer	137 (41)	82 (44)	0.072
	Breast cancer	65 (19)	27 (14)	
	Prostate cancer	48 (14)	40 (21)	
	Others	87 (26)	38 (20)	
Prophylactic administration	Natural vitamin D ± calcium	281 (82)	160 (86)	0.048
	Active vitamin D ± calcium	13 (4)	14 (7)	
	Calcium only	1 (1)	0 (0)	
	None	42 (12)	13 (7)	

Values in parentheses represent percentages

Multivariate logistic regression analysis revealed that the renal function, serum calcium (without albumin adjustment; Table 3, Model 1), and albumin-adjusted serum calcium (Table 3, Model 2) levels were significant independent risk factors. Note that all of these values identified as risk factors were pretherapeutic values.

**Table 3:** Multivariate logistic regression analysis to identify the risk factors for denosumab-induced hypocalcemia

Pretherapeutic factor (reference)	Category or unit	Model 1			Model 2		
		Odds ratio	95% CI	*P*-value	Odds ratio	95% CI	*P*-value
Serum calcium level	1 (mg/dL)	0.66	0.53–0.82	< 0.001			
Serum calcium level with albumin correction	1 (mg/dL)				0.61	0.48–0.77	< 0.001
Renal function classification (normal)	Mild	1.33	0.86–2.06	0.002	1.32	0.85–2.60	< 0.001
	Moderate	1.00	0.59–1.70		1.05	0.61–1.81	
	Severe	5.38	2.22–13.10		7.14	2.79–18.30	

CI: confidence interval

## Discussion

In this study, we reported the incidence of hypocalcemia by renal function in patients treated with denosumab for bone metastases of solid tumors and confirmed the importance of albumin adjustment in the assessment of hypocalcemia, independent of renal function. Furthermore, we identified low calcium levels prior to denosumab administration and severe renal dysfunction as risk factors for denosumab-induced hypocalcemia.

In a study including 55 patients, the incidence rate of hypocalcemia was higher in the 9 patients with severe renal dysfunction (GFR < 30 mL/min/1.73 m2) including hemodialysis than that in patients with moderate renal dysfunction to normal renal function (29.4% vs. 13.2%) [10]. In addition, in a study on 22 patients with cancer and severe renal dysfunction (Ccr < 30 mL/min), the incidence rate of hypocalcemia due to denosumab was 45%, and hypocalcemia of grade ≥ 3 severity occurred in 14% of the patients [15]. In the current study, we showed that the incidence of hypocalcemia was significantly higher among patients with severe renal dysfunction than among those with normal renal function, which is consistent with previous reports. However, the overall incidence rate of hypocalcemia in this study was 35.7% (187/524 patients); this is higher than that reported in a large-scale clinical trial (9.6%) [16], and in our opinion, is more reflective of the real-world clinical situation. Our findings confirm that patients with renal dysfunction need to be monitored carefully.

Figure 2 illustrates that the occurrence of grade ≥ 1 hypocalcemia was 37.4% in patients with mild renal dysfunction compared to 29.9% in those with moderate renal dysfunction; however, this difference was not statistically significant (*p* = 0.21). The lower prevalence of hypocalcemia in the moderate group, as opposed to the mild group, may be attributed to the administration of calcium or vitamin D supplements in 80–90% of these patients, as detailed in Table 1, potentially mitigating the onset of hypocalcemia.

Although phase I studies have shown that the pharmacokinetics and pharmacodynamics of denosumab are not affected by renal dysfunction, it has been reported that the risk of hypocalcemia is higher in patients with renal dysfunction [10]. While the kidneys are regulated by the parathyroid hormone and excrete calcium and phosphorus into the urine, they act as active vitamin D-producing organs. They are closely involved in calcium absorption in the intestinal tract. In other words, it is conjectured that abnormalities in calcium regulation due to CKD-associated mineral and bone disorders affect and cause hypocalcemia due to denosumab.

It is recommended that the albumin levels be measured when denosumab is administered. Approximately 50% of serum calcium is bound to serum proteins (principally albumin), and the remaining 50% is in the ionized form; (involved in several physiological processes) [17]. Low albumin levels lead to an increase in the proportion of free calcium ions. Thus, it is recommended that both the serum albumin and total serum calcium levels be measured simultaneously, and the total calcium level be adjusted using Payne’s formula [18] or another method [19]. Indeed, we identified a large discrepancy between the incidence of grade ≥ 1 hypocalcemia with and without albumin adjustment, which was 69.3% and 35.7%, respectively. However, albumin levels are often not measured in clinical practice, and although excluded from our analysis, in approximately 10% of patients in the collected data, albumin levels were not quantified.

Hypocalcemia severity in patients with severe renal dysfunction may be reduced by activated vitamin D. In patients with CKD, a decrease in blood vitamin D concentrations is observed [20, 21] and vitamin D activation is impaired, which is why reports suggest using activated vitamin D administration [22, 23]. In general, prophylactic replenishers used to prevent denosumab-induced hypocalcemia include a natural vitamin D preparation in addition to calcium; however, one study demonstrated that in patients with renal dysfunction, such replenishers might be inappropriate unless they contain activated vitamin D [24]. The same study addressed the relationship between the type of prophylactic replenisher used and the incidence of hypocalcemia and incorporated the kind of preventive treatment and identified risk factors into a multivariate logistic regression model [24]. Our study was unable to establish a significant impact of prophylactic agent type on hypocalcemia incidence, possibly due to the low proportion of patients using activated vitamin supplements (5%) and the high prevalence of patients (75%) receiving concurrent medications (Calcium 305 mg, Cholecalciferol 200IU, Magnesium 15 mg) for the prevention of denosumab-related hypocalcemia in bone metastases. Further studies are needed to clarify this aspect.

In the present multicenter study on patients with severe renal dysfunction, we identified low calcium levels and renal dysfunction as risk factors of hypocalcemia. There is no consensus among previous studies on cancer type being a risk factor; although not identified as a risk factor in our study as well, it remains possible that the incidence of hypocalcemia varies by the cancer type (*P* = 0.072, Table 2). Previous studies identified that risk factors for hypocalcemia include the type of cancer (hematologic malignancy and prostate, lung, and gastric cancers), poor performance status (PS), hospitalization, impaired kidney function, albumin-adjusted calcium level at baseline, elevated baseline bone turnover markers (urinary N-telopeptide of type I collagen and bone-specific alkaline phosphatase), higher baseline serum alkaline phosphatase levels, concomitant drug usage (vonoprazan and dexamethasone), and vitamin D deficiency at baseline [25–28]. Due to limitations on collection of patient data at institutions other than those administering denosumab in this retrospective observational study, bone metabolism markers and vitamin D levels were not measured and we could not analyze concomitant drug usage. Although PS and hospitalization affect the patients’ general condition, this study did not examine these aspects as well.

Hypocalcemia may not be the only problem in patients with severe renal dysfunction. The treatment continuation rate for denosumab differed significantly between patients with severe renal dysfunction and others (34.4% vs. 78.9%, *P* < 0.001). Furthermore, the retention rate was lower in patients with severe renal impairment, regardless of the presence of hypocalcemia (with and without hypocalcemia: 45.0% vs. 16.7%, *P* = 0.139). In other words, the denosumab continuation rate in patients with severe renal dysfunction was low regardless of the occurrence of hypocalcemia, and causes other than hypocalcemia may have affected the continuity of treatment; as we did not investigate the reasons for treatment discontinuation, obstacles to continuous therapy remain unclear.

Differences in patients’ backgrounds are a limitation of this study. We classified the patients into four groups according to their renal function level and observed that they tended to be older, weigh lesser, and be more affected by hypocalcemia with increasing severity of renal dysfunction. Although univariate logistic regression analysis did not identify age as a risk factor, older adult patients have reduced physiological organ functions and are more vulnerable to adverse events such as hemotoxicity from anticancer agents [29]. Consequently, we cannot rule out that denosumab-induced hypocalcemia may have been affected not only by renal function but also by age. In addition, while body weight was a covariate affecting the pharmacokinetic parameters of denosumab in population pharmacokinetic analyses during denosumab development, it has been concluded that at clinical doses, individual variation in pharmacokinetics due to body-weight differences do not cause significant differences in the pharmacological activity. Although albumin is necessary to obtain an accurate serum calcium level, denosumab is a monoclonal antibody and is unlikely to bind to plasma proteins. Therefore, albumin is presumed to not affect the drug’s action pharmacokinetically.

This study has several limitations. Firstly, we did not collect data regarding the symptoms caused by hypocalcemia or their subsequent treatment. Secondly, renal dysfunction encompasses a wide range of conditions, with the potential for varying incidence of hypocalcemia between acute kidney injury (AKI) and chronic kidney disease (CKD). The inability to delineate these differences was due to the lack of serum creatinine time-course data in our study. Thirdly, the collection of serum phosphorus data was inadequate, and alkaline phosphatase (ALP) measurements were not conducted, limiting our understanding of the etiology behind hypocalcemia development. Fourthly, we did not investigate the incidence of osteonecrosis of the jaw, a known side effect of bone-modifying drugs. Despite these limitations, our study sheds light on the prevalence of hypocalcemia in patients with severe renal dysfunction and highlights the role of albumin in this context.

When administering denosumab for bone metastases of solid tumors, we found that calcium monitoring with albumin adjustment is essential in patients with severe renal dysfunction to prevent overestimation of hypocalcemia. Furthermore, we found that this monitoring should be performed in all patients with cancer regardless of renal function level.

## Conclusions

Evaluating denosumab-induced hypocalcemia requires albumin adjustment, the incidence of which is high among patients with severe renal dysfunction. Reduced serum calcium levels and severely impaired renal function are associated with an elevated hypocalcemia risk, and denosumab should be administered with caution in these patients.

### Electronic supplementary material

Below is the link to the electronic supplementary material.

Supplementary Material 1

## Data Availability

The datasets used and/or analysed during the current study are available from the corresponding author on reasonable request.

## Abbreviations

Ccr: Creatinine clearance
CKD: Chronic kidney disease
IBW: Ideal body weight
PS: Performance status
