# Supplementary material for: Denosumab-induced hypocalcemia in patients with solid tumors and renal dysfunction: a multicenter, retrospective, observational study
Source: BMC Cancer. 2024 Feb 15;24:218. doi: 10.1186/s12885-024-11942-2 (PMC10870527; doi:10.1186/s12885-024-11942-2)
Supplement: Supplementary file 1 — Supplementary Material 1 [file 12885_2024_11942_MOESM1_ESM.docx]

Supplementary Table 1 Common Terminology Criteria for Adverse Events (CTCAE) v5.0

| CTCAE Term | Grade 1 | Grade 2 | Grade 3 | Grade 4 | Grade 5 |
| --- | --- | --- | --- | --- | --- |
| Hypocalcemia | Corrected serum calcium of <LLN - 8.0 mg/dL; <LLN - 2.0 mmol/L; Ionized calcium <LLN - 1.0 mmol/L | Corrected serum calcium of <8.0 - 7.0 mg/dL; <2.0 - 1.75 mmol/L; Ionized calcium <1.0 - 0.9 mmol/L; symptomatic | Corrected serum calcium of <7.0 - 6.0 mg/dL; <1.75 - 1.5 mmol/L; Ionized calcium <0.9 - 0.8 mmol/L; hospitalization indicated | Corrected serum calcium of <6.0 mg/dL; <1.5 mmol/L; Ionized calcium <0.8 mmol/L; life-threatening consequences | Death |

Supplementary Table 2 Univariate logistic regression analysis to identify risk factors for denosumab-induced hypocalcemia

| Factor (reference) | Category or unit | Odds ratio | 95% CI | *P*-value |
| --- | --- | --- | --- | --- |
| Sex (female) | Men | 0.79 | 0.549−1.140 | 0.205 |
| Age | 1 | 1.00 | 0.983−1.010 | 0.843 |
| Body weight | 1 | 1.01 | 0.990−1.020 | 0.489 |
| Serum albumin | 1 | 1.07 | 0.825−1.400 | 0.597 |
| Albumin-adjusted serum calcium level before administration of denosumab (mg/dL) | 1 | 0.68 | 0.537−0.853 | 0.001 |
|  |  |  |  |  |
| Creatinine clearance | 1 | 0.99 | 0.986−1.000 | 0.072 |
| Renal function classification (normal) | Mild | 1.27 | 0.820−1.960 | 0.008 |
|  | Moderate | 0.91 | 0.538−1.530 |  |
|  | Severe | 3.54 | 1.600−7.810 |  |
| Cancer type (breast cancer) | Lung cancer | 1.44 | 0.852−2.440 | 0.072 |
|  | Prostate cancer | 2.01 | 1.090−3.710 |  |
|  | Others | 1.05 | 0.584−1.890 |  |
| Prophylactic administration (natural vitamin D) | No supplemental medication | 0.54 | 0.283−1.040 | 0.094 |
|  | Active vitamin D | 1.89 | 0.867−4.120 |  |
|  | Others | 0.00 | 0.000−Inf |  |

CI: confidence interval.
